# Working Alliance in Online Cognitive Behavior Therapy for Anxiety Disorders in Youth: Comparison With Clinic Delivery and its Role in Predicting Outcome

**DOI:** 10.2196/jmir.1848

**Published:** 2012-06-28

**Authors:** Renee E E Anderson, Susan H Spence, Caroline L Donovan, Sonja March, Samantha Prosser, Justin Kenardy

**Affiliations:** ^1^School of PsychologyUniversity of QueenslandBrisbane, QLDAustralia; ^2^Griffith Health Research InstituteGriffith UniversityNathanAustralia; ^3^Centre of National Research on Disability and RehabilitationSchool of MedicineUniversity of QueenslandBrisbane, QLDAustralia

**Keywords:** Anxiety, online therapy, children, adolescents, working alliance

## Abstract

**Background:**

Substantial evidence exists that positive therapy outcomes are related to the therapist–client working alliance.

**Objectives:**

To report two studies that examined (1) the quality of the working alliance in online cognitive behavior therapy (CBT), with minimal therapist contact, for anxiety disorders in youth, and (2) the role of working alliance and compliance in predicting treatment outcome.

**Methods:**

Study 1 participants were 73 adolescents aged 12 to 18 years who met diagnostic criteria for an anxiety disorder, plus one or more of their parents. Participants were randomly assigned to clinic or online delivery of CBT, with working alliance being assessed for youth and parents after session 3. Study 2 participants were 132 children and adolescents aged 7 to 18 years who met diagnostic criteria for an anxiety disorder, plus one or more of their parents. Youths and parents participated in a minimally therapist-assisted online CBT program supported by brief, weekly emails and a single, short phone call.

**Results:**

Study 1 revealed a strong working alliance for both online and clinic CBT, with no significant difference in working alliance between conditions for adolescents (*F*_1,73 _= 0.44, *P *= .51, η_p_^2 ^= 0.006, Cohen *d *= 0.15). Parents also reported high working alliance in both conditions, although a slight but significantly higher working alliance in clinic-based therapy (*F*_1,70 _= 6.76, *P *= .01, η_p_^2 ^= 0.09, Cohen *d *= 0.64). Study 2 showed a significant and substantial decrease in anxiety symptoms following online therapy (*P *< .001 for all outcome measures). Adolescents improved significantly more in overall functioning when working alliance (beta = .22, *t*_79 _= 2.21, *P *= .03) and therapy compliance (beta = .22, *t*_84 _= 2.22, *P *= .03) were higher, with working alliance also predicting compliance (beta = .38, *F*_1,80 _= 13.10, *P *= .01). No such relationships were evident among younger children.

**Conclusions:**

Working alliance is important in determining clinical outcome for online treatment for anxiety among adolescents, with minimal therapist assistance, although this was not the case for younger children.

**Trial Registration:**

Australian New Zealand Clinical Trials Registry: ACTRN12611000900910; http://www.anzctr.org.au/trial_view.aspx?ID=343375 (Archived by WebCite at http://www.webcitation.org/674C4N3JJ)

## Introduction

Anxiety disorders among children and adolescents are prevalent and can result in significant deleterious consequences. Cognitive behavior therapy (CBT) has been shown to be highly efficacious in treating youth anxiety within a clinic-based setting [1,2] and, although only a handful of studies have examined the efficacy of an Internet-based treatment approach to youth anxiety, the results are encouraging and demonstrate similar remission rates to clinic-based CBT [3-5]. Yet, despite the impressive efficacy of CBT and regardless of the mode of delivery, not all young people respond positively to CBT for anxiety disorders. Indeed, approximately 43.5% of children continue to meet diagnostic criteria for a primary anxiety disorder following clinic-based CBT for an anxiety disorder [6], with similar rates evident in the few studies that have used either full or partial Internet delivery of treatment [3,4,7]. Exploring factors that might influence therapy outcome is therefore important, so that existing treatments can be improved upon and more effective treatment approaches can be developed. One factor that has been widely investigated with respect to its relationship to treatment outcome, at least within the adult, clinic-based treatment literature, is that of working alliance.

Working alliance, also referred to as therapeutic alliance, relates to the quality and nature of the therapist–client interaction, the collaborative approach toward the tasks and goals of treatment, and the personal bond or attachment that emerges in therapy [8,9]. There is substantial evidence that a strong working alliance between therapist and client is associated with positive psychotherapy outcomes [10,11]. A positive working alliance is proposed to result in increased motivation for and greater perseverence with task completion, reflecting an increased sense of optimism for a positive outcome and desire to please the therapist. In turn, greater completion of therapy tasks is proposed to result in greater acquisition of knowledge and skills, and a stronger sense of mastery [12]. In addition, a strong working alliance in CBT is proposed to facilitate positive cognitive change, provide disconfirming evidence of dysfunctional cognitions, and reinforce functional interpersonal behavior [13]. A recent meta-analytic review of 38 studies examining the association between working alliance and outcome in face-to-face child psychotherapy suggested a small but significant relationship with an average effect size of 0.14 [14]. Interestingly, and of relevance to the present studies, the effect size was smaller for adolescents (0.10) than for children (0.20), and also smaller for internalizing (0.10) than for externalizing disorders (0.22).

The emergence of online and computer-based delivery of psychological treatments has raised issues of whether a strong working alliance can be established in the absence of face-to-face contact with a therapist, and whether the quality of the online working alliance predicts treatment outcomes in this form of therapy. Indeed, online approaches have been criticized for their reduced capacity to establish a strong working alliance given the absence of nonverbal interpersonal cues, difficulty in monitoring client understanding of concepts, and limited ability to provide timely corrective feedback [15]. Despite the reservations expressed by some authors, however, empirical studies with adults have shown that a strong working alliance can indeed be established in online therapy and is comparable in strength with that of clinic-based treatment [16,17].

Regarding working alliance and outcome for online CBT for youth anxiety, only one study could be identified, and this was limited to a condition in which therapy was delivered primarily within the clinic but supplemented by computer-assisted technology [3]. The authors reported no significant differences on youth therapeutic alliance scores when therapy was partially computer delivered compared with a full clinic therapy format. It remains to be determined whether a strong working alliance can be produced when CBT for youth anxiety is delivered fully online with minimal therapist assistance and whether working alliance in this context predicts therapy outcome. Furthermore, given that treatment for anxiety disorders frequently involves parents, the quality of the working alliance must be considered from the perspective of both parties. The limited evidence available suggests that the working alliance involving the young person seems to be more important than that of the therapist–parent alliance in predicting outcome in clinic-based therapy [18]. However, it remains to be seen whether results would be similar for a treatment that is fully online with minimal therapist assistance.

The present studies sought not only to determine whether a sound working alliance could be developed during a completely online CBT treatment for youth anxiety and whether it could predict treatment outcome, but also to examine the mechanism of action through which working alliance might influence outcome. As noted above, the mechanism through which working alliance is suggested to affect treatment outcome is through greater treatment compliance [19,20]. Studies examining the association between therapy compliance and treatment outcome have produced mixed findings, possibly reflecting the varying definitions and methods of measurement that have been employed [21]. Karver and colleagues [21], however, found that both alliance and compliance predicted outcome for CBT among depressed adolescents, although the effects were direct, rather than mediated. Chu and Kendall [19], in a study involving clinic-based CBT for anxious youth, found that high compliance was associated with significantly better therapy outcomes at posttreatment. Whether compliance mediated the relationship between alliance and compliance, however, was not investigated in that study.

The relationship between alliance, compliance, and outcome does not appear to have been examined for anxious youth when CBT is fully delivered online. We proposed that alliance, through its effect on compliance, would be particularly important in predicting outcome in online therapy for anxious young people. With online therapy, young people are required to work more independently, and therefore a strong working alliance is hypothesized to be particularly important for maintaining motivation, enthusiasm, and effort to complete therapy tasks. Furthermore, we proposed that a similar set of predictive factors would exist for parents who are also required to participate in online treatment and contribute significantly to the therapy process.

The present research therefore had two main aims. First, we compared the relative strength of working alliance in online versus clinic delivery of CBT for anxiety in youth to determine whether it would be feasible to establish a strong working alliance between young people (and their parents) and their online therapist, despite minimal contact and the absence of face-to-face interaction. Although we hypothesized that the attempts to promote a strong working alliance from the online therapy would lead to a strong working alliance among young people and their parents, we did not expected that the working alliance would be as strong for online as for clinic-based therapy, given the absence of face-to-face interaction and its associated advantages.

Second, we examined whether the strength of the working alliance between the young people and their online therapist predicted therapy outcome for anxious youth following online delivery of CBT. Furthermore, we examined whether the proposed association between working alliance and outcome would be mediated by compliance with therapy tasks. Given the important role of parents in CBT for child anxiety [8], we hypothesized an equivalent set of predictive relationships between working alliance, compliance, and child therapy outcome for parents who participated in the online therapy.

Given the recent report by McLeod [14] that the alliance–therapy outcome relationship was stronger among younger children than among adolescents for face-to-face therapy, we also conducted subsidiary analyses by age given that the sample ranged in age from 7 to 18 years. As the nature and importance of working alliance may differ between online and face-to-face therapy, we did not formulate any firm directional hypotheses with respect to age. We also conducted subsidiary analyses to determine whether the effects differed by gender. Although McLeod did not find gender differences in the relationship between working alliance and outcome for face-to-face therapy, whether such differences exist in online treatment remains to be determined.

The research involved data from two overlapping samples and as such they are reported as separate studies.

## Study 1: Comparison of Working Alliance for Clinic and Internet Delivery

### Methods

#### Participants

Study 1 participants were 73 youths (45 female, 28 male) aged 12 to 18 years (mean 13.91, SD 1.56), plus one or more of their parents. We recruited families from metropolitan areas of Brisbane and Sydney, Australia, through referrals from guidance officers, mental health professionals, and self-referrals following media publicity. The majority of youths were born in Australia (n = 64, 88%) and lived with both biological parents (n = 58, 79%). Comparison of family incomes with the Australian Bureau of Statistics [22] census information indicated that, on average, participants came from middle- to high-income Australian families and were relatively well educated.

To participate, youths were required to have access to a computer and the Internet at home, to read at a minimum age level of 10 years, and to meet criteria for a principal diagnosis of separation anxiety disorder, generalized anxiety disorder, social phobia, or specific phobia. Referrals were excluded if the young person met criteria for a principal diagnosis of obsessive–compulsive disorder, oppositional defiant disorder, posttraumatic stress disorder, conduct disorder, dysthymia, or major depressive disorder with a clinician severity rating greater than 4 on the Anxiety Disorders Interview Schedule for Children and Parents (ADIS-C/P) [23], or had an intellectual handicap, learning disability, or pervasive developmental disorder. Referrals were also excluded if the young person was engaging in current self-harming behavior or was receiving treatment for anxiety elsewhere.

Youths and parents were administered the ADIS-C/P via telephone by a trained psychologist. All youths met the criteria outlined in the *Diagnostic and Statistical Manual of Mental Disorders*, fourth edition, text revision (DSM-IV-TR) for a primary anxiety disorder as determined by the ADIS-C/P (separation anxiety disorder: n = 12, 16%; generalized anxiety disorder: n = 34, 47%; social phobia: n = 22, 30%; specific phobia: n = 4, 6%; panic and agoraphobia: n = 1, 1%). Most (n = 68, 93%) youths had a secondary anxiety diagnosis. Following assessment, participants were randomly assigned to either the face-to-face therapy condition (n = 38) or the Internet condition (n = 35). Participants included those from the online and clinic conditions of a randomized controlled trial reported by Spence et al [5]. That study had also included a waitlist control condition and did not include the data relating to working alliance that are reported here.

#### Treatment Conditions

##### Clinic-Based Treatment

All clinic condition treatment sessions were conducted face-to-face with a therapist and followed the BRAVE program, which targets social phobia, generalized anxiety disorder, separation anxiety disorder, and specific phobia [24]. The program comprised 10 youth sessions and five parent sessions, each 1 hour long, as well as booster sessions at 1 and 3 months following treatment. Standard CBT anxiety management strategies were used, including psychoeducation, relaxation training, recognition of the physiological symptoms of anxiety, cognitive strategies of coping self-talk and cognitive restructuring, graded exposure, problem solving, and self-reinforcement. Parent sessions also taught anxiety management skills, in addition to parenting strategies to empower parents to help their child implement anxiety management skills.

Clinic sessions were conducted by registered psychologists at The University of Queensland, Griffith University, and Macquarie University psychology clinics. All clinicians received 2 days of training in use of the manualized clinic program, followed by weekly supervision.

##### Internet-Based Treatment

Online treatment participants completed BRAVE for Teenagers–ONLINE [7]. The BRAVE interventions have been described in detail elsewhere [4,25], and therefore the following contains only a brief overview. The content, length, and number of session activities in the Internet program replicate those of the clinic-based version. Sessions are designed to be engaging, interactive, and age appropriate. Eye-catching graphics, sounds, games, and quizzes are used to maintain the youths’ level of interest. Information is presented through interactive exercises and is followed by quizzes that check for correct understanding and provide personalized corrective or positive feedback through pop-up messages. The content of the intervention is designed to meet the developmental and cognitive level of youths, with age-appropriate scenarios, examples, and activities (example situations include school exams, job interviews, dating, and oral presentations).

The program is (minimally) therapist assisted, rather than self-help. Each family is assigned an online therapist (BRAVE trainer) who monitors their progress through the program and provides brief email feedback following each session. At no stage did any participants have face-to-face contact with their therapist and all other contact (ie, email or phone) was minimal. Clinician contact was restricted to brief, weekly emails and a short, 15-minute midprogram telephone call to assist in exposure hierarchy development. Most other contact with the online therapist was computer generated. Client responses to all session and homework activities were stored in an administrator section of the program and could be viewed by the therapist to guide the content of the weekly email. In addition, automated computer-generated emails were sent on behalf of the online therapist to congratulate participants for completing sessions, and personalized emails were sent to provide feedback about responses to quiz tasks. Personalized automated reminder emails were sent to advise when the next session would be available for completion or to provide prompts if the session was not completed by the due date. The first session also included a picture of the therapist and some brief biographical information about him or her, to which the client responded by providing information about him- or herself.

Youth and parent sessions were designed to be completed independently; however, there was no stipulation that parents could not help their child complete online sessions.

#### Measures

##### Anxiety Disorders Interview Schedule for Children–Parent and Child Version

The parent (ADIS-P) and a child (ADIS-C) interview schedules [23] were administered separately by trained clinicians. The ADIS determines the presence of anxiety disorders and other common disorders in children and adolescents. For each diagnosis obtained, the interviewer assigns a clinician severity rating ranging from 0 (no interference) to 8 (extreme interference). A rating of 4 (moderate interference) or more is considered to indicate a clinically significant diagnosis, with higher clinician severity rating scores representing more severe presentations of a disorder. The primary diagnosis was the diagnosis that was deemed to cause the most interference for the participant.

Interassessor reliability for the current study was determined from a random sample of interviews taken from 15% (n = 11) of families. This was conducted by two trained interviewers who were blind to the original diagnoses. Interassessor reliability was high for ADIS diagnoses, with a kappa value of 1 for the primary diagnosis and a correlation of .96 for the ADIS combined severity ratings.

##### Working Alliance Inventory-Short Form

The Working Alliance Inventory-Short Form (WAI-S) [26] is a 12-item scale, which includes three subscales: goal (agreement on the goals of therapy); task (agreement on how these goals will be achieved), and bond (the bond between participant and therapist). Participants were required to rate on a 7-point Likert scale from 1 (not at all true) to 7 (very true) the extent to which they believed each item was true of their relationship with their BRAVE trainer. Responses to all items were averaged to provide an overall working alliance score ranging from 1 to 7. Higher scores indicate a stronger working alliance (scores from 1 to 3 indicate a poor or negative relationship, 4 indicates a neutral position, and 5 to 7 indicate a good or positive therapeutic relationship). We obtained working alliance measures in both treatment conditions individually from youth participants and parents after they completed session 3. Working alliance was sampled early in treatment to minimize the potential confound between alliance scores and symptom improvement over the course of therapy [27].

### Study 1 Procedure

For the purpose of the study, we created separate versions of the WAI-S for youths and parents. We adjusted the wording of the items to account for both the clinic and online delivery of treatment, the reading level of younger clients (in the youth version), and the focus on the youth’s presenting problem in the parent version. The WAI-S has shown strong internal reliability [26,28,29] and factor structure in line with its theoretical model [30]. The WAI-S has also demonstrated adequate reliability and validity with youth populations [12,18].

The WAI-S was completed online by youths and parents, individually. Before using the measure in the present study, we examined whether the factor structure held up when administered online and in relation to therapist-assisted, online treatment. Consistent with prior research with adults in clinic-based therapy, it was predicted that confirmatory factor analysis would support a 3-correlated-factor model in which WAI-S items would load on intercorrelated factors relating to task agreement, goal agreement, and bond, with the covariation being explained to a large degree by a higher-order factor of working alliance in general [31]. The modified WAI-S was pilot tested with 137 clinically anxious children and adolescents (74 female, 63 male) aged between 7 and 18 years, plus one or more of their parents, participating in an online CBT intervention for anxiety. The results supported a single-factor model of working alliance for the young clients, with the comparative fit index = .96 and Tucker-Lewis Index = .97, demonstrating a good fit of the model (scores ≥ .95 indicate strong fit), and root mean square error approximation = 0.075 (90% confidence interval 0.05–0.10) and standardized root mean square residual = .03 (scores equal to or greater than .08 and .05, respectively, represent good data fit.). There were significantly correlated error terms between some items. A 3-correlated-factor model or higher-order model did not add significantly to the fit of the 1-factor model. Cronbach alpha for the youth WAI-S was .96. For parents, the data were well explained by a 2-factor model (bond and task/goal combined), loading onto a higher-order factor of working alliance, with comparative fit index = .95, Tucker-Lewis Index = .94, root mean square error approximation = 0.087 (90% confidence interval 0.06–0.11), and standardized root mean square residual = .07. Internal consistency was .94 for the parent WAI-S. Taken together, the results supported the use of the WAI-S total scores for both parents and youths in the online delivery of CBT for anxiety disorders and justified its use in the present study.

### Results

#### Pretreatment Comparison

Chi-square analyses showed no significant differences between the online and clinic conditions on gender (n = 73, χ^2^_1 _= 1.6, *P *= .24), parental marital status (n = 73, χ^2^_5 _= 6.1, *P *= .30), or child living situation (n = 73, χ^2^_3 _= 0.2, *P *= .98). Multivariate analysis of variance also revealed there were no significant differences between the clinic and online conditions for youth’s age, mother’s age, father’s age, education level of mother and father, and combined family income (Pillai *F*_6,66 _= 2.04, *P *= .07, η_p_^2 ^= .16). Chi-square analyses also showed no significant differences between the clinic and online conditions for the type of primary anxiety diagnoses (n = 73, χ^2^_5 _= 2.5, *P *= .78) or the number of comorbid nonanxiety disorders (n = 73, χ^2^_1 _= 0.3, *P *= .58). A multivariate analysis of variance revealed no significant differences in the severity of the primary anxiety diagnosis or the total number of anxiety diagnoses received (Pillai *F*_2,71 _= 0.24, *P *= .87, η_p_^2 ^= .01).

#### WAI-S Scores for Youths and Parents by Therapy Condition

**Figure 1:**
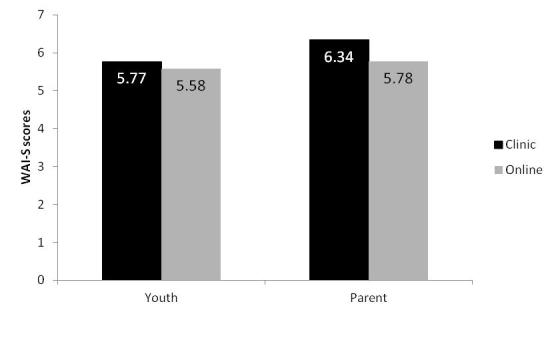
Mean Working Alliance Inventory-Short Form (WAI-S) scores for youths and parents in the face-to-face condition (clinic) and Internet condition (online).

Youth WAI-S scores ranged from 2.42 to 7.00 (mean 5.77, SD 1.20) for the clinic condition, and from 2.08 to 7.00 (mean 5.58, SD 1.34) for the online condition. There were no significant differences between the groups (*F*_1,73 _= 0.44, *P *= .51, η_p_^2 ^= .006, Cohen *d *= 0.15), and youths in both groups reported strong working alliance (see Figure 1). Clinic condition parent WAI-S scores ranged from 4.00 to 7.00 (mean 6.34, SD 0.67), and online condition parent WAI-S scores ranged from 3.17 to 7.00 (mean 5.78, SD 1.06). Although both online and clinic condition parents rated the working alliance with their therapist as positive and strong, those in the clinic condition rated alliance with their face-to-face therapist significantly higher than parents in the online condition (*F*_1,70 _= 6.76, *P *= .01, η_p_^2 ^= .09, Cohen *d *= 0.64). No significant correlations were found between youth and parent WAI-S scores in either the clinic (*r *= .09, *P *= .60) or online (*r *= .20, *P *= .22) treatment conditions.

## Study 2: Prediction of Online Therapy Outcome From Working Alliance and Compliance

### Methods

#### Participants

Participants in study 2 were 132 children and adolescents who met criteria for an anxiety disorder plus at least one parent, who had completed a minimum of three sessions of online-delivered CBT (the point at which the working alliance measure was administered) and for whom outcome data were obtained at 6-month follow-up. Participants in study 2 included the online group from study 1, but we supplemented the sample to include additional adolescents and expanded the age range to include youth aged from 7 to 12 years. This extended sample increased the statistical power to enable examination of predictive factors using regression analyses and the testing of moderating variables such as age and gender. Given the extended age range and addition of further participants to those reported in study 1, the participant characteristics for study 2 will be reported in full. There were 70 female and 62 male participants, aged 7 to 18 years (mean 12.12, SD 2.50), plus one or more of their parents. The majority were born in Australia (n = 119, 90.1%) and were living with both biological parents (107, 81.1%). On average, the participants came from middle- to high-income Australian families and were relatively well educated. The inclusion and exclusion criteria were identical to those in study 1. All children and adolescents met DSM-IV-TR criteria for a primary anxiety disorder, including separation anxiety disorder (n = 28, 21%), generalized anxiety disorder (n = 53, 40%), social phobia (n = 41, 31%), or specific phobia (n = 10, 8%), according to the ADIS-C/P. The mean clinician severity rating for the principal anxiety diagnosis was 5.95 (SD 0.76), and the mean number of anxiety disorders was 2.87 (SD 1.44). In total, 117 participants (88.6%) had at least two diagnoses.

#### Study 2 Procedure

Procedures were identical to those outlined for study 1, with the exception that participants were restricted to those participating in online therapy (with no clinic comparison group). Outcome was evaluated 6 months after the 12-week treatment period. The follow-up assessment included an ADIS interview completed with each child or adolescent and one parent, and the clinician-rated Children’s Global Assessment Scale (CGAS) [32]. The follow-up interviews were completed by trained clinicians who were blind to participants’ treatment condition. Treatment effectiveness was measured at 6-month follow-up to provide a stable and longer-term indicator of outcome, with the key indicator for assessing the prediction of outcome being the CGAS rating. We selected the CGAS rather than clinician ratings of primary anxiety disorder severity (clinician severity rating) or parent/youth questionnaire measures, as it provided a more comprehensive indication of overall improvement made by the child or adolescent, rather than improvement made for only the principal anxiety disorder or a subjective response to symptom checklists.

#### Measures

The WAI-S and the ADIS-C/P described for study 1 were also used in study 2. We administered additional youth and parent self-report measures of anxiety in study 2 to determine whether youths improved from pretreatment to 6-month follow-up after completing the online CBT intervention, and whether there was variability in outcome in order to justify the examination of predictors.

#### Primary Outcome Measure

##### Children’s Global Assessment Scale

The CGAS [32] provides a single global rating of functioning, assigned to the youth by the independent interviewing clinician, on a scale of 0 to 100, where lower scores indicate poorer functioning. A rating is given based on the child’s or adolescent’s most impaired level of general functioning for the specified time period by selecting the lowest level that describes his or her functioning on a hypothetical continuum of health–illness, benchmarked against anchor points in a descriptive glossary. The CGAS has demonstrated sufficient sensitivity in detecting treatment outcomes for child anxiety (eg, March et al [4]). It has shown high interrater reliability with a correlation ranging from .59 to .90 [32-35].

Interassessor reliability for the current study was determined from a random sample of audiotaped interviews taken from 15% (n = 20) of families, conducted by two trained, independent interviewers who were blind to the original diagnoses. Interassessor reliability was high for the CGAS ratings (*r *= .94).

#### Secondary Outcome Measures

##### Child Behavior Checklist-Revised

Parents completed the Child Behavior Checklist (CBCL) [36], a psychometrically sound measure of behavioral problems for young people aged between 4 and 18 years. Parents were asked to rate whether an item applied to their child, using a 3-point scale (0 = not true; 1 = somewhat or sometimes true; 2 = very true or often true). We used only the internalizing scale in the present study.

##### Spence Children’s Anxiety Scale-Parent Version and Child Version

We used the Spence Children’s Anxiety Scale-Parent Version (SCAS-P) and Child Version (SCAS-C) [37,38] to measure child and adolescent anxiety symptoms. The SCAS-P and SCAS-C assess symptoms relating to separation anxiety, social phobia, obsessive–compulsive disorder, panic–agoraphobia, generalized anxiety, and fears of physical injury according to symptom clusters represented by the DSM-IV-TR. Children and parents rate on a 4-point scale how frequently the child experiences a particular event, with higher scores representing higher levels of anxiety. We used only the total scores in the study’s analyses. The SCAS-C has demonstrated sound psychometric properties, with an internal reliability coefficient of .92 for the total score, and its factor structure has been confirmed in several studies [37-39]. The parent version of the SCAS also demonstrates sound psychometric properties, with high internal consistency for total anxiety scores (alpha = .89) [40]*.*

#### Compliance With Internet Sessions

Program compliance was operationalized as the percentage of therapy sessions and homework tasks completed by the participant by the 6-month follow-up. As described above, online sessions comprised multiple activities. During each session, participants were required to respond to exercises and quizzes that involved providing responses or typing answers into the program. These responses were stored electronically in individual file logs, accessed through the administrative section of the online program. The total number of tasks to be completed included all session activities where the participant was able to enter a response into the program, from sessions 1 through 10 for youths and 1 through 6 for parents (or five sessions for parents of adolescents, as material from the six sessions was condensed into five sessions for parents of this age group).

Responses to tasks were scored as either 0 = not completed, or 1 = completed. For an item to be considered completed and to receive a score of 1, the participant must have attempted the activity with a meaningful response. A trained researcher reviewed each participant’s log file to determine whether the participant had attempted to complete each task in a meaningful way and assigned a score of 0 or 1 accordingly. The total number of tasks completed was divided by the total number of tasks for the entire intervention to obtain a percentage compliance score.

A second trained researcher selected a random sample of 15% (n = 20) of log files and coded them using the same guidelines for scoring. Independent interrater agreement on total compliance scores was high (kappa = 0.975).

#### Internet Therapy Program

The online intervention in the current study was outlined above for study 1. However, given the age range of the young people involved, we created two versions of the site so that the reading age required, illustrations, examples, and graphics were developmentally appropriate. For example, the site for the younger children used a cartoon character (Brave Buddy) who represented the coping model, whereas the adolescent version used cartoon teenaged coping models to illustrate the concepts.

#### Analyses

We conducted a series of standard and hierarchical multiple regression analyses to test whether the variables of interest were associated with treatment outcome or treatment compliance. Analyses to test mediation were also used where appropriate as outlined by Baron and Kenny [41]. Baseline CGAS scores were entered into each hierarchical equation at step 1 of all analyses in the prediction of CGAS at 6-month follow-up, to control for pretreatment severity.

### Results

#### Treatment Outcome

The first step was to determine whether changes in anxiety over time were significant, with sufficient variation in outcome to justify examining prediction of response to treatment. As these analyses were not central to the purpose of the study, we discuss them only briefly.

#### Changes in Anxiety Following Treatment

Table 1 and Table 2 present data regarding treatment outcome and analyses. At 6-month follow-up, significant improvements were evident for all outcome measures, including diagnostic criteria, overall functioning on the CGAS, and reductions in internalizing and anxiety symptoms over time for CBCL, SCAS-C, and SCAS-P. Taken together, the results indicated significant improvements in emotional wellbeing over time, but with variation in response, justifying the examination of predictors of treatment outcome.

#### Compliance With Internet-Based Treatment Tasks

The mean number of sessions recorded as completed by children and adolescents was 8.86 (SD 1.90) out of 10 sessions. Parents completed an average of 5.74 sessions (SD 0.66) out of 6 in the child program and 4.76 sessions (SD 0.56) out of 5 in the adolescent program. In terms of session tasks, youths completed an average of 85% (SD 20.22, range 26% to 100%, 25 to 95 tasks) of available tasks and parents completed an average of 89% (SD 12.32, range 27% to 100%, 15 to 55 tasks) of tasks available to them.

#### Working Alliance

The mean child and adolescent WAI-S at the end of session 3 was 5.85 (SD 1.09) with a range from 2.08 to 7.00, and the mean parent WAI-S was 6.07 (SD 0.76) with a range from 2.92 to 7.00. On average, both parents and youths formed a strong working alliance with their online therapist.

#### Correlations Between Alliance, Compliance, and Outcome Measures

There was a significant and positive correlation between youth alliance and parent alliance (*r *= .31, *P *< .001), such that higher levels of parent alliance were associated with higher levels of youth alliance. This finding differed from that of study 1, in which youth alliance and parent alliance were not significantly correlated. In study 2, this can be explained by a much higher correlation between parent and child WAI-S scores among 7- to 11-year-old children (*r *= .51, *P *< .001) than among the adolescents in the sample (*r *= .26, *P *< .001).

**Table 1:** Treatment outcomes for children and adolescents with at least one anxiety disorder assessed in study 2.

Measure	Pretreatment		6-month follow-up		*F *value	df (hypothesis, error)	η_p_^2^
	Mean	SD	Mean	SD			
CSR^a^	5.96	0.76	2.44	1.96	413.17***	1,130	.76
CGAS^b^	49.82	4.95	70.80	11.09	554.93***	1,130	.81
Number of diagnoses	2.87	1.44	0.82	1.16	265.34***	1,130	.67
Youth SCAS-C^c^	39.59	17.14	22.04	13.63	177.34***	1,112	.61
Parent SCAS-P^d^	32.33	14.04	18.01	8.49	139.85***	1,116	.55
CBCL^e^	21.19	7.96	10.87	7.48	146.99***	1,117	.60

^a ^Clinician severity rating.

^b ^Children’s Global Assessment Scale.

^c ^Spence Children’s Anxiety Scale-Child Version.

^d ^Spence Children’s Anxiety Scale-Parent Version.

^e ^Child Behavior Checklist.

**Table 2:** Children and adolescents free of primary diagnosis and any diagnosis at pretreatment and 6-month follow-up.

Type of diagnosis	Pretreatment		6-month follow-up		χ^2^	df
	n	%	n	%		
Free of primary diagnosis	132	0	87	66.4	85.01***	1,131
Free of any diagnosis	132	0	70	53.4	68.01***	1,131

***P < .001.

A positive relationship was also found between parent compliance and youth compliance (*r *= .53, *P *< .001). Youth alliance measured after session 3 was significantly and positively associated with youth compliance by 6-month follow-up (*r *= .30, *P *< .001). Parent alliance was not significantly associated with parent compliance or youth compliance. Youth alliance and parent alliance, and youth compliance and parent compliance were not significantly correlated with the outcome (CGAS at 6-month follow-up). As expected, baseline CGAS was significantly correlated with CGAS at follow-up (*r *= .40, *P *< .001).

Demographic variables (age of youth, household income, and mother’s and father’s education) were not significantly correlated with any of the independent variables (parent alliance or youth alliance, and parent compliance or youth compliance), the baseline measure of severity (CGAS), or the dependent variable (CGAS at 6-month follow-up), except for mother’s education, which was positively and significantly correlated with youth alliance (*r *= .21, *P *< .05).

#### Alliance Predicting Outcome

To examine whether alliance predicted outcome, we performed a hierarchical multiple regression. Pretreatment CGAS scores were entered on the first step to control for the effects of baseline severity. Youth alliance was entered on the second step. The overall model was significant at step 2 (*F*_2,128 _= 12.22, *P *< .001). Baseline CGAS was a significant predictor of CGAS at 6-month follow-up (beta = .40, *t*_129 _= 4.93, *P *< .001). When we examined the unique effects of youth alliance at step 2, it was not a significant predictor of CGAS at 6-month follow-up (beta = .08, *t*_129 _= 0.95, *P *= .34). Similarly, when we examined the unique effects of parent alliance at step 2, it was not a significant predictor of CGAS at 6-month follow-up (beta = .12, *t*_129 _= 1.49, *P *= .14).

#### Alliance Predicting Compliance

The analysis revealed that youth alliance significantly and positively predicted youth compliance (beta = .30, *F*_1,127 _= 12.32, *P *= .001) and accounted for approximately 9% of the variance in compliance scores. However, parent alliance did not significantly predict parent compliance (*F*_1,126 _= 0.36, *P *= .55).

#### Compliance Predicting Outcome

Pretreatment CGAS scores were entered on the first step to control for the effects of baseline severity and youth compliance entered on the second step. The overall model was significant (*F*_2,128 _= 13.76, *P *< .001). When we examined the unique effects of youth compliance at step 2, it was not a significant predictor of CGAS at 6-month follow-up (beta = .08, *t*_129 _= 0.95, *P *= .34). Similarly, when we examined the unique effects of parent compliance at step 2, it did not significantly predict CGAS at 6-month follow-up (beta = .14, *t*_129 _= 1.74, *P *= .08). Given the absence of direct effects between alliance and compliance with outcome, it was not therefore appropriate to examine a mediation model.

#### Gender as a Moderator

We examined gender as a possible moderating variable in the relationship between youth alliance and youth compliance with outcome at 6-month follow-up. A moderated hierarchical regression analysis was performed with baseline CGAS entered first to control for pretreatment severity. Youth alliance and gender were entered into the regression at step 2. The interaction term was entered on step 3. However, the interaction between youth alliance and gender did not significantly predict outcome (beta = .01, *t*_129 _= .16, *P *= .87). We then performed a moderated hierarchical regression analysis for the interaction between youth compliance (centered) and gender in predicting outcome, but this was also not significant (beta = .09, *t*_129 _= 1.11, *P *= .27).

#### Age as a Moderator

Youth age was then examined as a moderating variable in the prediction of CGAS outcome, based on age as a continuous variable. The centered variables, youth alliance and age, were entered into the regression analysis at step 2 after controlling for baseline CGAS. We found a main effect for age (beta = .17, *t*_129 _= 2.01, *P *= .05), such that older age was associated with significantly better outcome on CGAS at 6 months. At step 3, the interaction term for the two variables was added. The overall model was significant (*F*_4,123 _= 8.54, *P *< .001). There was no main effect for youth alliance (beta = .08, *t*_129 _= .97, *P *= .33), and the main effect for age remained significant. The interaction between youth alliance and age significantly predicted CGAS outcome (beta = .17, *t*_129 _= 2.08, *P *= .04), which accounted for 3% of the variance in outcome.

**Figure 2:**
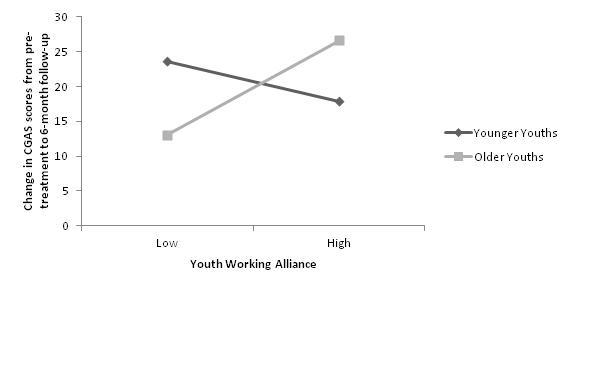
Conditional effects of youth age on the relationship between youth working alliance and change in child global functioning, as measured by the Children’s Global Assessment Scale (CGAS), from pretreatment to 6-month follow-up. Younger/older age represents -/+1 SD (2.5 years) below/above the mean (12.12 years).

We conducted simple slopes analyses for younger (1 SD below the mean) and older (1 SD above the mean) youths, and low (1 SD below the mean) and high (1 SD above the mean) alliance, to aid interpretation. Simple slopes analyses (see Figure 2) indicated that for adolescents there was a significant and positive relationship between alliance and change in CGAS scores from pretreatment to 6-month follow-up, such that a stronger alliance was associated with a greater positive change in CGAS and thus a better treatment outcome (B = 2.73, SE = 1.15, *t*_128 _= 2.37, *P *= .02). For younger youths, the relationship between alliance and change in CGAS scores from pretreatment to 6-month follow-up was not significant (B = –1.11, SE = 1.33, *t*_128 _= –0.83, *P *= .41).

Change in CGAS scores aided in the interpretation of the simple slopes analyses and figures.

 We then tested age as a moderating variable in the relationship between parent alliance and outcome at 6-month follow-up. The centered variables of parent alliance and age of youth were entered into the regression analysis at step 2, after controlling for baseline CGAS at step 1, followed by the interaction term for the two centered variables at step 3. The overall model was significant (*F*_4,121 _= 8.96, *P *< .001). There was no significant main effect for parent alliance (beta = .11, *t*_125 _= 1.40, *P *= .17) or age (beta = .16, *t*_125 _=1.93, *P *= .06). However, there was a significant interaction between parent alliance and age of youth (beta = .19, *t*_125 _=2.36, *P *= .02) that accounted for 4% of the variance.

**Figure 3:**
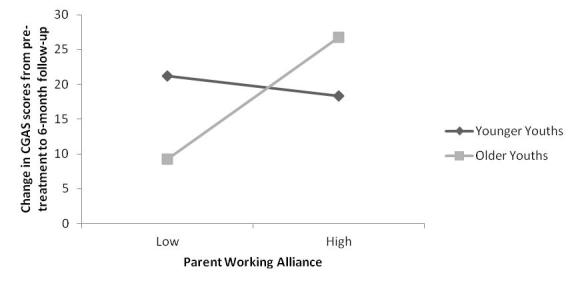
Conditional effects of youth age on the relationship between parent working alliance and change in child global functioning, as measured by the Children’s Global Assessment Scale (CGAS), from pretreatment to 6-month follow-up. Younger/older age represents -/+1 SD (2.5 years) below/above the mean (12.12 years).

Simple slopes analyses (see Figure 3) indicated that for older youths there was a significant and positive relationship between parent alliance and change in CGAS scores from pretreatment to 6-month follow-up, such that a stronger parent alliance was associated with a greater positive change in CGAS and thus a better treatment outcome (B = 4.11, SE = 1.55, *t*_125 _= 2.65, *P *< .01). For younger youths, the relationship between parent alliance and change in CGAS scores from pretreatment to 6-month follow-up was not significant (B = –0.86, SE = 1.59, *t*_125 _= –0.54, *P *= .59).

 We then tested age as a moderating variable in the relationship between youth compliance and outcome at 6-month follow-up. The centered variables of youth compliance and youth age were entered into the regression analysis at step 2, after controlling for baseline CGAS at step 1, followed by the interaction term for the two centered variables at step 3. The overall model was significant (*F*_4,126 _= 10.03, *P *< .001). There was no significant main effect for youth compliance (beta = .06, *t*_128 _= 0.75, *P *= .46) or age (beta = .08, *t*_128 _= 0.92, *P *= .36). However, there was a significant interaction between youth compliance and age (beta = .25, *t*_128 _= 3.02, *P *= .003) that accounted for 6% of the variance.

**Figure 4:**
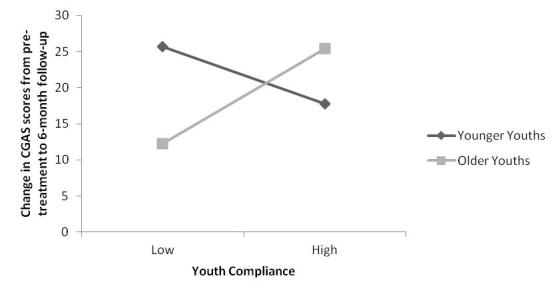
Conditional effects of youth age on the relationship between youth compliance and change in child global functioning, as measured by the Children’s Global Assessment Scale (CGAS), from pretreatment to 6-month follow-up. Younger/older age represents -/+1 SD (2.5 years) below/above the mean (12.12 years).

Simple slopes analyses (see Figure 4) indicated that for older youths there was a significant and positive relationship between youth compliance and change in CGAS scores from pretreatment to 6-month follow-up, such that a higher youth compliance was associated with a better treatment outcome (B = 1.79, SE = 0.50, *t*_125 _= 3.55, *P *< .001). For younger youths, the relationship between compliance and change in CGAS scores from pretreatment to 6-month follow-up was not significant (B = –0.48, SE = 0.52, *t*_125 _= –0.93, *P *= .35).

 Lastly, we tested age as a moderating variable in the relationship between parent compliance and outcome on the CGAS at 6-month follow-up. The centered variables of parent compliance and age of youth were entered into the regression analysis at step 2, after controlling for baseline CGAS at step 1, followed by the interaction term for the two centered variables at step 3. The overall model was significant (*F*_4,126 _= 8.46, *P *< .001). There was no significant main effect for parent compliance (beta = .14, *t*_128 _=1.70, *P *= .09) or age (beta = .08, *t*_128 _= 0.87, *P *= .39). The interaction between parent compliance and age did not significantly predict outcome (beta = .14, *t*_128 _=1.63, *P *= .11).

#### Age Differences Between Variables

To inform possible explanations for the differential pattern of results by age, we divided the sample into younger children (aged 7 to 11 years) and adolescents (aged 12 to 18 years). Table 3 presents the means, standard deviations, and regression analyses for adolescents and children. Adolescents had significantly lower CGAS scores at baseline than the younger group (*F*_1,130 _= 8.05, *P *< .01, η_p_^2 ^= .06) but no significant differences in CGAS at 6-month follow-up (*F*_1,129 _= 0.02, *P *= .90, η_p_^2 ^= .00). Working alliance at session 3 was slightly but significantly lower among adolescents than among children (*F*_1,128 _= 4.01, *P *= .05, η_p_^2 ^= .03).

 Parents’ ratings of alliance measured after session 3 were not significantly different for the two age groups (*F*_1,126 _= 0.22, *P *= .64, η_p_^2 ^= .00). However, parents of adolescents were significantly more compliant with the treatment program by 6-month follow-up than were parents of children (*F*_1,131 _= 6.87, *P *= .01, η_p_^2^= .05).

**Table 3:** Means, standard deviations, and regression analyses for alliance, compliance, and CGAS scores compared between children and adolescents.

Measure	Children (7–11 years)		Adolescents (12–18 years)		*F*	df	*P *value	η_p_^2^
	Mean	SD	Mean	SD				
Youth alliance	6.10	.80	5.71	1.21	4.01	1,128	.05	.03
Parent alliance	6.06	.76	6.07	.77	0.22	1,126	.64	.00
Youth compliance	84.09	20.98	84.89	19.90	0.05	1,131	.83	.00
Parent compliance	85.00	16.11	91.68	12.72	6.87	1,131	.01	.05
Baseline CGAS^a^	51.47	4.77	48.98	4.86	8.05	1,130	.01	.06
Follow-up CGAS	70.64	10.79	70.89	11.32	0.02	1,129	.90	.00
Δ CGAS^b^	19.17	10.27	22.00	10.07	2.35	1,131	.13	.02

^a ^Children’s Global Assessment Scale.

^b ^Change in CGAS from pretreatment to 6-month follow-up.

#### Examination of Mediational Model for Adolescents

Given the findings indicating that age significantly moderated the relationships between youth alliance and outcome and between youth compliance and outcome, and that compliance and outcome and alliance and outcome were significantly associated in the simple slopes analyses for older youths (aged 12 to 18 years), we considered it appropriate to examine the mediation model with the older age group separately. Table 4 summarizes the results.

First, youth alliance significantly predicted CGAS at 6-month follow-up (beta = .22, *t*_79 _= 2.21, *P *=.03) and accounted for 5% of the variance. Second, compliance significantly predicted CGAS at 6-month follow-up (beta = .22, *t*_84 _= 2.22, *P *= .03) and accounted for 5% of the variance. Third, adolescent alliance significantly and positively predicted adolescent compliance (beta = .38, *F*_1,80 _= 13.10, *P *= .01), accounting for approximately 14% of the variance in compliance scores.

In the final step of testing, the mediation model examined whether the significant relationship between adolescent alliance and outcome decreased in significance or became nonsignificant when adolescent compliance was entered into the equation. Pretreatment CGAS scores were entered at the first step to control for the effects of baseline severity. Adolescent compliance and adolescent alliance were entered at the second step. The overall model was significant (*F*_3,77 _= 9.84, *P *< .001); however, when adolescent compliance was controlled for, adolescent alliance no longer significantly predicted CGAS scores at 6-month follow-up (beta = .17, *t*_79 _= 1.56, *P *= .12). Because adolescent compliance was not significant at step 2, despite adolescent alliance becoming nonsignificant, a mediation model was not supported. Partial mediation effects were also tested. Bootstrapping results for the indirect effect [42] confirmed that no mediation or partial mediation existed, with the 95% bias-corrected bootstrap confidence interval based on 5000 bootstrap samples (–1.12 to 1.40, with a point estimate of 0.09).

**Table 4:** Summary of mediational analyses for the adolescent group.

Group			*R* ^2^	Adjusted *R* ^2^	*R* ^2 ^∆	*F *∆	df	Beta	sr^2^
**Adolescent alliance predicting adolescent compliance at 6-month follow-up**									
	Step 1								
		Adolescent alliance	.14	.13	.14	13.10	1,80	.38**	.14
**Adolescent alliance predicting CGAS^a ^ scores at 6-month follow-up**									
	Step 1								
		Baseline CGAS scores	.21	.20	.21	20.89	1,79	.51***	.24
	Step 2								
		Baseline CGAS scores	.26	.24	.05	4.89	2,78	.50***	.24
		Adolescent alliance						.22*	.05
**Adolescent compliance predicting CGAS scores at 6-month follow-up**									
	Step 1								
		Baseline CGAS scores	.21	.20	.21	21.68	1,82	.49***	.24
	Step 2								
		Baseline CGAS scores	.25	.24	.05	4.91	2,81	.48***	.24
		Adolescent compliance						.22*	.05
**Adolescent compliance as a mediator in the relationship between adolescent alliance and outcome on CGAS at 6-month follow-up**									
	Step 1								
		Baseline CGAS scores	.25	.24	.25	13.30	1,79	.52***	.26
	Step 2								
		Baseline CGAS scores	.28	.25	.02	2.42	3,77	.51***	.26
		Adolescent compliance						.16	.02
		Adolescent alliance						.17	.02

^a ^Children’s Global Assessment Scale.

*P < .05; **P < .01; ***P < .001.

 Thus, for the adolescents, although both alliance and compliance significantly predicted treatment outcome at follow-up when entered separately, and alliance significantly predicted compliance, compliance was not found to significantly mediate the relationship between adolescent alliance and CGAS scores at 6-month follow-up. Rather, both alliance and compliance had direct effects on treatment outcome.

## Discussion

The present research confirms that a strong working alliance can be established for both parents and their children during online therapy, despite the absence of face-to-face contact and with only minimal therapist contact through email and a brief phone call. Indeed, from the perspective of adolescents, there was no significant difference in the strength of the working alliance between online and clinic delivery of CBT for youth anxiety. Although parents reported a significantly greater working alliance from clinic-based than from online therapy, the difference was extremely small and the working alliance in online therapy was still strong. Firm conclusions cannot be drawn about the determinants of the strong working alliance; however, it seems likely that the personalized pop-ups, automated feedback, and emails were beneficial in achieving this goal. The research also demonstrated the construct validity of the WAI-S as an indicator of the quality of therapist–client relationship in minimal therapist-assisted online treatment for young people and their parents and the strong internal reliability of the measure. The strong psychometric properties justified its use in the subsequent analyses.

The positive treatment response demonstrated in study 2 following online therapy was consistent with previous research involving either partial or full Internet or computer delivery of CBT interventions with young people [3,4,7,25]. In study 2, 66% (n = 87) of treated children and adolescents were free from their primary anxiety diagnosis by 6-month follow-up, which is consistent with much of the clinic-based literature in this area [6]. However, also consistent with clinic-based treatment of youth anxiety disorders was the finding that a considerable proportion of youth retained at least one clinical diagnosis of anxiety after treatment (n = 62, 47%). It was important, therefore, to investigate factors that may predict treatment outcome and that could potentially be influenced in order to produce better outcomes.

Although working alliance and therapy compliance were not found to significantly predict treatment outcome for the total sample, a consistently different pattern of results emerged according to the age of the young person. For younger children, there were no significant relationships between working alliance, compliance, and outcome. In contrast, for adolescents a stronger working alliance for both the youth and the parent predicted greater positive change in CGAS scores by 6-month follow-up. Similarly, higher youth compliance predicted more positive changes in CGAS scores from pretreatment to 6-month follow-up for adolescents. Also, consistent with our hypotheses, greater working alliance among adolescents predicted greater therapy compliance. However, contrary to predictions, compliance was not found to mediate the alliance–outcome relationship, with the effects being direct rather than mediated. Interestingly, this finding is consistent with that of Karver and colleagues [21], who found that both alliance and compliance predicted outcome for CBT among depressed adolescents, and that the effects were direct, rather than mediated. Whether mediated or not, the results emphasize the importance of a strong working alliance and compliance with therapy tasks in leading to better outcomes in the adolescent group.

It is important to consider why positive associations between alliance, compliance, and outcome should be evident for adolescents but not for the younger age group. Both age groups, in general, formed a strong working alliance with their online therapist, and compliance was equivalent across age groups. Outcome was slightly stronger among the adolescent group, although this may reflect their slightly poorer level of adjustment at baseline. The parents of the adolescents also showed slightly, but significantly, higher levels of therapy compliance than the parents of the younger children. One can only speculate about some of the reasons why working alliance and compliance were more important in predicting outcome for adolescents. Perhaps with younger children, anxiety problems are more transient and the rate of improvement may be faster, leading to earlier cessation of therapy tasks because improvements are already evident. Future research needs to track symptom severity more frequently during therapy in order to examine rates of change, as it is likely that the relationship between compliance and outcome is a complex one and there may be many reasons why people do not comply with treatment tasks. Another point to consider is that the nature of the relationship between young people changes with age. Younger children may be more reliant on their parents for support during therapy, and the quality of their perceived relationship with their online therapist may be less important to determining their level of treatment compliance or outcome. Adolescents, on the other hand, tend to be more independent from their parents and may place greater value on their online therapist for support and encouragement. This proposition is consistent with the finding of little correlation between parent and adolescent WAI-S scores with their therapist, whereas the association between younger children’s and their parents’ WAI-S scores was much stronger. We must also question whether younger children were as free to express their personal views of the WAI-S or were influenced in their judgments by parental guidance. Perhaps their understanding or view of alliance is different from that of adolescents. Clearly, the findings emphasize how important it is to consider age differences in this type of research. The sample size in study 2 was sufficiently large to enable examination of age effects, something that has not been possible in studies with smaller sample sizes and insufficient power.

Several limitations must be considered for the current studies. First, it is possible that the inclusion of both qualitative *and *quantitative measures of compliance may have provided a more comprehensive assessment of compliance than the quantitative-only analyses in the current studies. It is noteworthy, however, that for adolescents, compliance significantly predicted CGAS at 6-month follow-up, suggesting that more sensitive qualitative measurements of compliance may be most relevant in younger cohorts and their parents. Second was the inability to examine in detail how working alliance, compliance, and anxiety changed over time on a session-by-session basis. This means that it is not possible to separate cause–effect relationships. Finally, it is not clear whether the results are specific to online CBT, with minimal therapist support, for child anxiety problems or whether they will generalize to online therapy alone, or indeed to clinic-based treatment. These remain questions for future research.

In summary, the present research demonstrated that a strong working alliance can be formed by parents and youths completing an online CBT intervention for anxiety. Indeed, we observed no significant differences in ratings of alliance in a clinic and online intervention for youths. Age significantly moderated the relationship between alliance, compliance, and outcome. For adolescents, in keeping with hypotheses, both working alliance and compliance positively and significantly predicted treatment outcome, and alliance significantly predicted compliance. However, compliance did not mediate the relationship between alliance and outcome. Younger children also tended to rate alliance very strongly, although alliance and compliance were not found to predict clinical outcomes. Taken together, the results highlight the importance of a strong client–therapist relationship in online therapy and support the use of online interventions for anxiety-disordered children and adolescents.

## Abbreviations

ADIS-C/P: Anxiety Disorders Interview Schedule for Children and Parents
CBT: cognitive behavior therapy
CGAS: Children’s Global Assessment Scale
DSM-IV-TR: Diagnostic and Statistical Manual of Mental Disorders, fourth edition, text revision
SCAS-C: Spence Children’s Anxiety Scale-Child Version
SCAS-P: Spence Children’s Anxiety Scale-Parent Version
WAI-S: Working Alliance Inventory-Short Form

## References

[1] Kendall PC, Flannery-Schroeder E, Panichelli-Mindel SM, Southam-Gerow M, Henin A, Warman M (1997). Therapy for youths with anxiety disorders: a second randomized clinical trial. J Consult Clin Psychol.

[2] Wood JJ, Piacentini JC, Southam-Gerow M, Chu BC, Sigman M (2006). Family cognitive behavioral therapy for child anxiety disorders. J Am Acad Child Adolesc Psychiatry.

[3] Khanna MS, Kendall PC (2010). Computer-assisted cognitive behavioral therapy for child anxiety: results of a randomized clinical trial. J Consult Clin Psychol.

[4] March S, Spence SH, Donovan CL (2009). The efficacy of an internet-based cognitive-behavioral therapy intervention for child anxiety disorders. J Pediatr Psychol.

[5] Spence SH, Donovan CL, March S, Gamble A, Anderson RE, Prosser S, Kenardy J (2011). A randomized controlled trial of online versus clinic-based CBT for adolescent anxiety. J Consult Clin Psychol.

[6] Cartwright-Hatton S, Roberts C, Chitsabesan P, Fothergill C, Harrington R (2004). Systematic review of the efficacy of cognitive behaviour therapies for childhood and adolescent anxiety disorders. Br J Clin Psychol.

[7] Spence SH, Holmes J, Donovan CL (2006). BRAVE for Teenagers-ONLINE: An Internet Based Program for Adolescents With Anxiety.

[8] Kazdin AE, Whitley M, Marciano PL (2006). Child-therapist and parent-therapist alliance and therapeutic change in the treatment of children referred for oppositional, aggressive, and antisocial behavior. J Child Psychol Psychiatry.

[9] Bordin ES, editor (1975). The Working Alliance: Basis for a General Theory of Psychotherapy.

[10] Baldwin SA, Wampold BE, Imel ZE (2007). Untangling the alliance-outcome correlation: exploring the relative importance of therapist and patient variability in the alliance. J Consult Clin Psychol.

[11] Horvath AO, Bedi RP (2002). The alliance. Norcross JC, editor. Psychotherapy Relationships That Work: Therapist Contributions and Responsiveness to Patients.

[12] Kazdin AE, Marciano PL, Whitley MK (2005). The therapeutic alliance in cognitive-behavioral treatment of children referred for oppositional, aggressive, and antisocial behavior. J Consult Clin Psychol.

[13] Waddington L (2002). The therapy relationship in cognitive therapy: a review. Behav Cogn Psychother.

[14] McLeod BD (2011). Relation of the alliance with outcomes in youth psychotherapy: a meta-analysis. Clin Psychol Rev.

[15] Rochlen AB, Zack JS, Speyer C (2004). Online therapy: review of relevant definitions, debates, and current empirical support. J Clin Psychol.

[16] Knaevelsrud C, Maercker A (2007). Internet-based treatment for PTSD reduces distress and facilitates the development of a strong therapeutic alliance: a randomized controlled clinical trial. BMC Psychiatry.

[17] Cook JE, Doyle C (2002). Working alliance in online therapy as compared to face-to-face therapy: preliminary results. Cyberpsychol Behav.

[18] Hawley KM, Garland AF (2008). Working alliance in adolescent outpatient therapy: youth, parent and therapist reports and associations with therapy outcomes. Child Youth Care Forum.

[19] Chu BC, Kendall PC (2004). Positive association of child involvement and treatment outcome within a manual-based cognitive-behavioral treatment for children with anxiety. J Consult Clin Psychol.

[20] Shirk SR, Karver M (2003). Prediction of treatment outcome from relationship variables in child and adolescent therapy: a meta-analytic review. J Consult Clin Psychol.

[21] Karver M, Shirk S, Handelsman JB, Fields S, Crisp H, Gudmundsen G, McMakin D (2008). Relationship processes in youth psychotherapy: measuring alliance, alliance-building behaviors, and client involvement. J Emot Behav Disord.

[22] Anonynous (2007). Australian Bureau of Statistics.

[23] Silverman WK, Albano AM (1996). The Anxiety Disorders Interview Schedule for Children for DSM-IV.

[24] Donovan CL, Holmes J, Spence SH (2006). BRAVE for Teenagers: A Program for Adolescents With Anxiety.

[25] Spence SH, Donovan CL, March S, Gamble A, Anderson R, Prosser SJ, Kercher A, Kenardy J (2008). Online CBT in the treatment of child and adolescent anxiety disorders: issues in the development of BRAVE-ONLINE and two case illustrations. Behav Cogn Psychother.

[26] Tracey TJ, Kokotovic AM (1989). Factor structure of the Working Alliance Inventory. Psychol Assess.

[27] Feeley M, DeRubeis RJ, Gelfand LA (1999). The temporal relation of adherence and alliance to symptom change in cognitive therapy for depression. J Consult Clin Psychol.

[28] Hanson WE, Curry KT, Bandalos DL (2002). Reliability generalization of working alliance inventory scale scores. Educ Psychol Meas.

[29] Leibert T, Archer J (2006). An exploratory study of client perceptions of Internet counseling and therapeutic alliance. J Ment Health Counsel.

[30] Busseri MA, Tyler JD (2003). Interchangeability of the Working Alliance Inventory and Working Alliance Inventory, Short Form. Psychol Assess.

[31] Horvath AO, Greenberg LS (1989). Development and validation of the Working Alliance Inventory. J Couns Psychol.

[32] Shaffer D, Gould MS, Brasic J, Ambrosini P, Fisher P, Bird H, Aluwahlia S (1983). A children's global assessment scale (CGAS). Arch Gen Psychiatry.

[33] Bird HR, Canino G, Rubio-Stipec M, Ribera JC (1987). Further measures of the psychometric properties of the Children's Global Assessment Scale. Arch Gen Psychiatry.

[34] Dyrborg J, Larsen FW, Nielsen S, Byman J, Nielsen BB, Gautrè-Delay F (2000). The Children's Global Assessment Scale (CGAS) and Global Assessment of Psychosocial Disability (GAPD) in clinical practice--substance and reliability as judged by intraclass correlations. Eur Child Adolesc Psychiatry.

[35] Rey JM, Starling J, Wever C, Dossetor DR, Plapp JM (1995). Inter-rater reliability of global assessment of functioning in a clinical setting. J Child Psychol Psychiatry.

[36] Achenbach TM (1991). Manual for the child behavior checklist/4-18 and 1991 profile.

[37] Spence SH (1997). Structure of anxiety symptoms among children: a confirmatory factor-analytic study. J Abnorm Psychol.

[38] Spence SH (1998). A measure of anxiety symptoms among children. Behav Res Ther.

[39] Muris P, Schmidt H, Merckelbach H (2000). Correlations among two self-report questionnaires for measuring DSM-defined anxiety disorder symptoms in children: the Screen for Child Anxiety Related Emotional Disorders and the Spence Children's Anxiety Scale. Pers Individ Dif.

[40] Nauta MH, Scholing A, Rapee RM, Abbott M, Spence SH, Waters A (2004). A parent-report measure of children's anxiety: psychometric properties and comparison with child-report in a clinic and normal sample. Behav Res Ther.

[41] Baron RM, Kenny DA (1986). The moderator-mediator variable distinction in social psychological research: conceptual, strategic, and statistical considerations. J Pers Soc Psychol.

[42] Hayes AF (2009). Beyond Baron and Kenny: statistical mediation analysis in the new millennium. Commun Monogr.

